# Recombinant therapeutic proteins degradation and overcoming strategies in CHO cells

**DOI:** 10.1007/s00253-024-13008-6

**Published:** 2024-01-29

**Authors:** Shao-Lei Geng, Xiao-Jie Zhao, Xi Zhang, Ji-Hong Zhang, Chun-Liu Mi, Tian-Yun Wang

**Affiliations:** 1 International Joint Research Laboratory for Recombinant Pharmaceutical Protein Expression System of Henan, Xinxiang Medical University, Xinxiang, 453003 Henan China; 2 School of Basic Medical Sciences, Xinxiang Medical University, Xinxiang, 453003 Henan China; 3 Henan Engineering Research Center for Biopharmaceutical Innovation, Xinxiang Medical University, Xinxiang, 453003 Henan China; 4 School of Pharmacy, Xinxiang Medical University, Xinxiang, 453003 Henan China

**Keywords:** Chinese hamster ovary cells, Recombinant therapeutic proteins, Protein quality, Degradation, Bioprocess engineering

## Abstract

**Abstract:**

Mammalian cell lines are frequently used as the preferred host cells for producing recombinant therapeutic proteins (RTPs) having post-translational modified modification similar to those observed in proteins produced by human cells. Nowadays, most RTPs approved for marketing are produced in Chinese hamster ovary (CHO) cells. Recombinant therapeutic antibodies are among the most important and promising RTPs for biomedical applications. One of the issues that occurs during development of RTPs is their degradation, which caused by a variety of factors and reducing quality of RTPs. RTP degradation is especially concerning as they could result in reduced biological functions (antibody-dependent cellular cytotoxicity and complement-dependent cytotoxicity) and generate potentially immunogenic species. Therefore, the mechanisms underlying RTP degradation and strategies for avoiding degradation have regained an interest from academia and industry. In this review, we outline recent progress in this field, with a focus on factors that cause degradation during RTP production and the development of strategies for overcoming RTP degradation.

**Key points:**

*• The recombinant therapeutic protein degradation in CHO cell systems is reviewed.*

*• Enzymatic factors and non-enzymatic methods influence recombinant therapeutic protein degradation.*

*• Reducing the degradation can improve the quality of recombinant therapeutic proteins.*

## Introduction

Compared with prokaryotic cells, yeast cells, and insect cell expression systems, mammalian cell expression systems have post-translational modifications (PTMs) similar to human cells. Its expression products are closest to natural higher biological proteins in terms of molecular structure, physicochemical properties, and biological functions. Therefore, the mammalian cell expression system is an important platform for recombinant therapeutic proteins (RTPs) production at present (Lu et al. 2020). The mammalian expression system mainly includes expression vector, serum-free medium, and cell line. Among them, the mammalian cell lines include non-human cell lines and human cell lines, and the commonly used non-human mammalian cell is Chinese hamster ovary (CHO) cell (Ha et al. 2023). Although non-human mammalian cells have safety and efficacy, RTPs expressed are easy to cause immunity in the human body, resulting in the elimination of foreign RTPs by the circulatory system, due to the different PTMs, especially the difference in glycosylation from human cells. Human embryonic kidney (HEK) 293 cells are widely used as human cell lines for recombinant protein drug production (Borsi et al. 2023). Mammalian cell lines have the integrated capabilities to express and secrete RTPs, and a large number of cell lines with suitable growth characteristics that could be obtained from various tissues and species. Considering the production capacity, biological activity, cost, and safety of the proteins of interest (POIs), the optimal mammalian cell expression system was selected.

Currently, nearly 70% of approved RTPs are produced from CHO cell system (Kang et al. 2023; Stolfa et al. 2018; Wang and Guo 2020). After continuous domestication and modification of CHO cells, subtypes such as CHO-K1, CHO-S, CHO-DBX11, and CHO-DG44 have been obtained (MacDonald et al. 2022b). At present, methotrexate selection system based on dihydrofolate reductase (DHFR) (Wang et al. 2021) and methionine sulfoxide selection system based on glutamine synthetase (GS) (Teixeira et al. 2022) are the main CHO cell expression systems that are widely used in industry. Compared with other host cells, CHO cells are less susceptible to human viruses, can adapt to industrial large-scale serum-free suspension expression, and express proteins with full PTMs similar to their natural state. Moreover, the genes of interest (GOIs) are easily stably integrated into the CHO cell genome and show the ability of efficient gene replication and expression levels (Raab et al. 2022; Tihanyi and Nyitray 2020). With the progress and development of medium optimization and cell culture technology, the expression levels of RTPs in CHO cells (Gbico, #A13696-01) were significantly increased and some monoclonal antibody titers exceeded 10 g/L under the conditions of fed-batch culture and perfusion culture (Handlogten et al. 2018; Srila et al. 2023; Xu et al. 2023). By 2020, more than 70 monoclonal antibodies (mAbs) are already on the market and global sales will reach $125 billion (Liang et al. 2023; Urquhart 2021). Biopharmaceutical market approval from 2014 to 2018 showed that more than 80% of newly approved biopharmaceuticals were produced with CHO cells, and that number had risen to 89% until 2022 (Walsh and Walsh 2022). Therefore, the CHO cell expression system has become a powerful recombinant protein drug production platform.

## Degradation of RTPs

The quality of a therapeutic protein refers to its physical, chemical, and biological properties that enable them to function properly (Ha et al. 2022). Quality properties such as glycosylation, aggregation, charge variant, and degradation determine the activity, efficiency, safety, pharmacokinetics, and pharmacodynamics of RTPs (Nguyen and Zimmer 2023; Torkashvand and Vaziri 2017). However, the biggest obstacles encountered when using mammalian cells such as CHO cell lines is the degradation of POIs by peptides expressed and secreted in the cell culture medium, which is also one of the important factors affecting the quality of antibodies (Wen et al. 2018).

Degradation, mainly includes fragmentation, hydrolysis, and cleavage, refers to the destruction of protein structure in the production of RTPs under the action of enzymes or non-enzymes, resulting in the heterogeneity of glycosylation, charge, etc., which are crucial to the stability, half-life, immunogenicity, and biological function of the drug (Nguyen and Zimmer 2023; Torkashvand and Vaziri 2017). The heterogeneity caused by degradation will also affect the yield of the obtained proteins, and also increase the burden on downstream purification processes (Bryan et al. 2021; Budge et al. 2021).

RTP degradation could occur at multiple stages, from production to storage management. The formation of protein degraders would negatively affect its impact on product yield, quality, safety, and effectiveness. The primary, secondary, and tertiary structures of proteins, glycosylation patterns, and susceptibility to physical and chemical damage are the main factors that affect protein degradation. It was found that the intermediates in the antibody degradation process cannot bind to the Fcg receptor, nor to the complement protein C1q, and thus, lacking immune function, and resulting in antibody-dependent cell antibody-mediated cytotoxicity (ADCC) and complement-dependent cytotoxicity (CDC) (Ha et al. 2022; Vlasak and Ionescu 2011). The affinity of tumor-associated proteases to intact antibodies is significantly higher than that of monomer antibodies, resulting in the ineffectiveness of anti-tumor antibodies against certain diseases. Some incorrectly folded proteins or oxidation-damaged proteins are preferred to degrade into amino acids and reused to maintain protein homeostasis. In the process of antibody production, the correctly assembled monoclonal antibody (mAb) and bispecific antibody (BsAb) also degrade at the same time with the heteropolymers degradation, significantly reduced the yield and biological activity of the antibody drug (Qin et al. 2022). With advances in protein engineering, the demands for the production of more complex proteins, such as bispecial antibodies and fusion proteins, has increased dramatically. These engineered proteins often contain modified sequences, or disulfide bonds, which make them easier to aggregate and degrade (Wang et al. 2019).

In our previous studies, some strategies have been committed to improve the expression of recombinant proteins in mammalian cells (Life Technologies, #A11557-01), and screen the efficient expression vector elements (Li et al. 2022a; Wang and Guo 2020; Wang et al. 2023; Yang et al. 2022), construct the cell line for enhancing transgenic expression and long-term stability (Jia et al. 2018) and optimize serum-free cell medium (Li et al. 2022b; Xu et al. 2023) and other new optimization strategies in cell culture were also explored (Lu et al. 2023; Yang et al. 2022). With the increasing demands for RTPs, more attentions should be paid to the quality attributes of RTPs besides increasing the yields. However, the degradation of RTPs during production is one of the important factors affecting the quality. Therefore, higher quality production of RTPs needs further research.

Here, we mainly discussed various factors and mechanisms affecting RTP degradation in the industrial production process of CHO cells, and reviewed the relevant effective strategies to inhibit the degradation at the present stage.

## Degradation types of RTPs

### Enzymatic degradation

At present, protein degradation in CHO cells is mainly dependent on endoplasmic reticulum-associated protein degradation (ERAD), ubiquitin–proteasome system, and autophagy pathways, including macroautophagy, microautophagy, and chaperon-mediated autophagy. Increased RTP production tends to break through the limitations of ER folding capacity and trigger activation of the ERAD pathway, thereby degrading excess abnormally folded/processed proteins in ER (Hussain et al. 2014; Urquhart 2021). Most proteasome substrates, including key regulatory proteins, are proteins with short half-lives, which are involved in cell division, signaling, and transcription (Shiber and Ravid 2014). Proteases, both intracellular and extracellular, are involved in the enzymatic degradation of proteins. This degradation process is regulated by the oligosaccharide portion of glycoproteins, as well as by glycosidases, including sialase secreted in the medium.

mAbs have several sites in the conserved hinge region that are sensitive to enzyme and non-enzyme degradation, and can undergo enzymatic degradation, such as papain, resulting in reduced biological function (Ha et al. 2022). In clinical settings, several proteases associated with inflammation, tumor invasion, metastasis, and bacterial infection have the ability to split IgG. These proteases primary cut IgG in the hinge, including matrix metalloproteinases (MMPs), which includes stromal lysozyme 1(MMP-3) and metalloelastase (MMP-12) (both of which are cleaved between Pro232 and Glu233), maternal mitogen (MMP-7) (cleaved between Lys234 and Ser235), cathepsin G, staphylococcus aureus glutamylendopeptide I (GluV8) (cleaved in Glu2 cleavage occurs between Ser233 and Ser234) and streptococcus pyogenes degrading enzymes (cleavage between Gly236 and Gly237). These proteases have a synergistic effect on the gradual cleavage of IgG proteins (Schauer et al. 2018; Vlasak and Ionescu 2011).

Some of the proteases secreted by CHO cells, unlike sialidase(s) and glycosidase (s), which are passively released into the medium upon cell death, are secreted during cell culture in bioreactors. Thus, their activities cannot be minimized by maintaining high cell viability and cause product fragmentation (and subsequent aggregation), while others can lead to immunogenic responses in patients (Alhuthali and Kontoravdi 2022). Serine proteases, elastase, collagenase, and plasminogen activators are not only passively released in cell culture upon cell death, but also many proteases are actively secreted into the culture medium, and membrane-bound proteases are in direct contact with RTPs physiologically, so their presence is independent of cell viability or the duration of culture and their degradation of RTPs was particularly significant (Hu et al. 2022; Mols et al. 2005).

### Non-enzymatic degradation

Non-enzymatic degradation is caused by physical or chemical changes. Since proteins maintain their structural stability in a certain range of pH, osmotic pressure, and temperature, changes in these parameters would destroy the protein structure and cause degradation (Ha et al. 2022). Shah et al. found that light oxidized two key methionine residues in the Fc region of protein and analyzed the effects on the structure, stability, aggregation, and function of mAb (Shah et al. 2018). Therapeutic proteins may undergo a variety of chemical degradation processes, including oxidation, deamidation, isomerization, hydrolysis, deglycosylation, racemization, disulfide bond breaking and formation, Maillard reaction, and β-elimination (Le Basle et al. 2020). Oxidative deamidation is the most common chemical degradation process of mAb, which may result in changes in their physical properties such as hydrophobicity, charge, secondary and/or tertiary structure, and lower thermodynamic or kinetic barriers to unfolding. This may make the product susceptible to polymerization and other chemical modifications that alter the binding affinity, half-life, and efficacy of the product (Gupta et al. 2022). These modifications may alter biological properties and therapeutic activity, can cause immunogenicity, and may lead to further degradation.

Protein fragmentation is a widespread degradation, such as non-enzymatic fragmentation of the peptide backbone of mAbs. The easily fragmental sites in polypeptide chains are determined by various factors, and there are some bonds in specific side chains. Studies have shown that the hinge region of IgG and Fc fusion proteins is unstable. A set of key cutting sites on the hinge of human IgG1, and some specific regions show increased sensitivity to hydrolysis or cleavage in the sequence Ser219-Cys220-Asp221-Lys222-Thr223-His224-Thr225-Cys226, especially in Asp221, and Lys222. These sites were identified for non-enzymatic cleavage (Dorai and Ganguly 2014; Vlasak and Ionescu 2011).

### Other degradations

In addition to the above factors, there are other unknown reasons that cause protein degradation. The aeration rate of RTP production in bioreactors is closely related to the removal of CO_2_ and the generation of foam. Too low aeration rate will lead to rapid accumulation of CO_2_ in the medium and decrease pH (Pereira et al. 2018), and acidic pH-induced isomerization of Asp221 and His mediated-hydroxyl radical formation, both of which contribute to the cleavage of adjacent peptide bonds, and antibody degradation. Increasing the concentration of PF-68 negatively affects filtration performance, reduces the ability of RTPs to pass through the filter, and prolongs product retention in the bioreactor, which increasing protein degradation and product quality (Wei et al. 2023; Xu et al. 2017).

## Overcome strategies to RTP degradation

### Co-expressed genes

Eukaryotic cells have evolved a variety of strategies to control the balance of synthesis and degradation of intracellular and extracellular proteins. The function of molecular chaperons has traditionally been associated with protein folding, assembly, aggregates, and degradation. It is mainly divided into *heat shock proteins* (HSPs), redox enzymes, and other special chaperones (Freilich et al. 2018). HSPs are highly conserved chaperones responsible for the refolding or degradation of folded and misfolded proteins. *Glucose-regulated protein 94* (GRP94), interacts with immunoglobulin heavy chains, light chains, and insulin-like proteins, is upregulated during ER stress to help fold proteins and guide ERAD (Samy et al. 2021). Growing pieces of evidence show that molecular chaperones play important roles in the degradation pathway and are the main regulators of protein degradation. Hsp40 and Hsp90 can participate in the regulation of activation-induced cytidine deaminase degradation (Kohler and Andreasson 2020).

The introduction of exogenous genes into CHO cells will enhance the incorrect incorporation of amino acids into the primary sequence of POIs, resulting in heterogeneity. However, overexpressed genes can induce an UPR in RTP production, leading to the overloading and expansion of ER proteins, and inhibiting recombinant protein degradation in cells. Cotransfection of CHO-derived transcription factor *Yin-Yang 1* (YY1) increased the volume productivity of Ri-tuximab in transient expression systems (CHO cell line, ATCC: CCL-61, CTL-11397) (Tastanova et al. 2016), confirming that YYI was involved in the reduction of ERAD of folding intermediates (Hussain et al. 2014), as well as autophagy and lysosomal mediated protein degradation processes (Kim et al. 2013). Differential transcriptomic sequencing revealed that ERAD genes (*Ubx*, *Derlin*) and ubiquitin ligase complex genes (*UBXN8*, *HERPUD1*, *DNAJB9*) were critical for the elevated load of aggregation-prone antibodies and polypeptides in the ER (Table 1) and overexpressed in Ab1-secreting cells. Antibody titers can be effectively reduced through protein degradation, thereby reducing aggregation (Barzadd et al. 2022).

**Table 1:** Gene overexpression strategy of RTPs degradation in CHO cells

Cell Type	Condition	Outcome	References
CHO-K1	GRP94	Modulate protein fold and ERAD	Samy et al. 2021
	Hsp40, Hsp90	Participate in activation-induced cytidine deaminase degradation	Kohler and Andreasson 2020
CHO-K1	*Ubx*, *Derlin*, *UBXN8*, *HERPUD1*, *DNAJB9*	Inhibition aggregation and induce degradation	Barzadd et al. 2022

ERAD, endoplasmic reticulum-associated protein degradation

### Gene knockout

CRISPR-Cas9 gene editing technology has been used in CHO cell line engineering to alter the expression of many endogenous genes and produce modified cell lines with the desired phenotype (Kheirandish et al. 2023; Rahimi et al. 2023), to improve productivity, growth characteristics and glycosylation, as well as enhance the long-term stability of transgenic expression, and eliminate problematic host cell proteins (HCPs) (Glinsek et al. 2023).

Cell line engineering can be used to reduce or eliminate HCPs, reducing the need for additional purification steps (Table 2). Some proteins encoded by mammalian host cells are difficult to remove during downstream processing and have potential negative effects on product quality (Goey et al. 2018; Kol et al. 2020; Luthra et al. 2021). Many endogenous proteases, active in cell culture, are capable of degrading recombinant expressed proteins and secreted peptides, which may show very strong site specific or have a wide range of substrates. Due to differences in the expression of endogenous proteases in various cell lines, the degradation effects of proteases on RTPs can be changed through cell line screening (Liu et al. 2019; Zhu 2012). *Lipoprotein lipase* (LPl) has been found to be one of the most common difficult-to-remove HCPs. LPl degrades polysorbate, an excipient commonly used in final drug formulations, negatively affecting the stability of RTPs (Glinsek et al. 2023). Therefore, LPl is an ideal candidate for gene knockout. Consistently, Lpl expression was eliminated and polysorbate degradation decreased by targeted gene mutation; however, there was no substantial effect on cell viability (Chiu et al. 2017).

**Table 2:** Gene editing strategy of RTP degradation in CHO cells

Cell type	Condition	Outcome	Reference
CHO-K1	*Lipoprotein lipase* KO	Improved stability of polysorbate 20 (up to 57%) and polysorbate 80 (up to 47%), without significant impact cell viability	Chiu et al. 2017; Glinsek et al. 2023
CHO-K1	*Matriptase-1* KO	Reduced or no proteolytic degradation activity toward a panel of recombinantly expressed proteins	Laux et al. 2018
CHO-S	*MGAT1* KO	Limits glycosylation to Man5 and earlier intermediates,	Byrne et al. 2018
CHO-K1	*The complement component 1 protease (C1s)*	Inhibit gp120 protein proteolytic clipping by a serine protease	Li et al. 2019
CHO-K1	*C1s*, *MGAT1* KO	Led to production of unclipped gp120 protein with high mannose glycans	Li et al. 2020; Li et al. 2019
CHO-K1	*Anxa-2* and *Cathepsin D* KO	Led to complete elimination of corresponding HCPs in cell lysates without affecting cell growth and viability	Fukuda et al. 2019
CHO-K1	*Cathepsin D* KO	Led to almost complete elimination of the associated mAb degradation	Dovgan et al. 2021; Lim et al. 2018
CHO-DUXB11	*CpD*	CpD KO prevent C-terminal lysine of IgG from cleavage	Hu et al. 2016
CHO-S	*Timp1*, *Lgals3bp*, *Bgn*, *Nid1.1*, *Nid1.2*, *Ctsd*, *Tinagl1*, *Erp29*, *Aga*, *Lgmn*, *Gpr56*, *Yeats2*, *Sparc*, *Lpl*	6-, 11-, and 14-HCPs KO led to non-negligible impact on protein production as well as degradation	Kol et al. 2020

*KO*, knockout; *MGAT1*, acetylglucosaminyltransferase; *CpD*, carboxypeptidase D

*Matriptase-1*, a serine protease expressed in CHO-K1 cells, is a major protease involved in the degradation of RTPs. When matriptase-1 was knocked out using transcriptional activator-like effector nucleases (TALENs), there was little or no degradation of the protein incubated in the supernatant of the knockout cell culture, whereas degradation of the target protein reached 50–100% in wild-type CHO-K1 cells (Laux et al. 2018).

Some studies have shown that the degree of glycosylation of RTPs is directly related to sensitivity to proteases, because the presence of more glycan portions reduces protease access to proteolytic cleavage sites in the protein (Raju and Scallon 2006). When expressed in CHO cells, several candidate HIV subunit vaccines are easily degraded or cleavage due to the lack of important sugar-dependent epitopes on virions, such as glycoprotein 120 (gp120) and the monomer subunit of HIV-1 envelope protein (Env) (Robinson 2018). Therefore, the study found that by using the CRISPR/Cas9 gene editing system to inactive *Mannosyl-(α-1,3-)-Glycoprotein-β-1,2-N-Acetylglucosaminyltransferase* (MGAT1) gene, thus to limit the glycosylation of Man5 and early intermediates and effectively reduce the degradation of recombinant subunit vaccines (Byrne et al. 2018). Consistent with the results, Li et al. showed that knockout of the *complement component 1 protease* (C1s) in CHO cell lines using CRISPR/Cas9 can eliminate proteolytic activity against recombinant expressed gp120. Furthermore, C1s^−/−^ and MGAT1^−/−^ double-knocked CHO cell lines (ATCC:CCL-61) can efficiently produce untailored gp120 and enrich multiple mannose-5 glycans necessary for widespread neutralization of mAb binding (Li et al. 2020, 2019).

Some proteases, such as cladin, enolase, peroxidoredoxin 1, actin, and phospholipid transfer proteins, exhibit proteolytic activity which leads to protein fragmentation. *Cathepsin D* (CSTD) is a lysosomal aspartic protease that interacts with hydrophobic motifs (LYY, LY, or YY) in mAbs and is difficult to be removed. Knockout experiments on CSTD hydrolase alone showed almost elimination of the associated proteolytic enzyme degradation remaining during the purification of mAbs (Dovgan et al. 2021; Lim et al. 2018). Fukuda et al. found that *annexin A2* (Anxa2), a member of the calcium-dependent phospholipid-binding protein family, involved in cell growth and signal transduction pathways. Anxa2 and CSTD genes were knocked out simultaneously, reducing the risk of overcontamination and degradation of RTPs by host cells (RCB-0285) without affecting normal cell growth (Fukuda et al. 2019).

The heterogeneity of C-terminal lysine levels in recombinant therapeutic antibodies is due to the hydrolysis of endogenous *Carboxypeptidase D* (CpD), which is the only enzyme responsible for C-terminal lysine cleavage in CHO cells. When using CRISPR/Cas9 technology to knock out CpD, C-terminal lysine cleavage in CpD knockout cells completely disappeared (Hu et al. 2016). In a recent study, using transcriptomics techniques to analyze and guide the experiments, more than 6-, 11-, and 14 genes were knocked out through CRISPR/Cas9 gene editing, creating a “clean” CHO cell by deleting the most common HCP, making the difficult to remove during downstream processing and proteins that have a potential negative impact on product quality are removed. Moreover, the degree of degradation and fragmentation of RTPs is significantly reduced (Kol et al. 2020).

### Vector optimization

The common approach for GOIs to avoid protein degradation is sequence optimization, base mutation, fragment deletion, and fusion with peptide or protein tags at the N or C-terminal to eliminate amino acid motifs that are prone to degradation (Dorai et al. 2011; Robert et al. 2009; Strohl 2018). IGF-1 is a hormone with the molecular structure similar to insulin, and the production of a mutated human insulin-like growth factor-1 precursor (hIGF-1Ea mut) in CHO cells leads to poor cell growth and low productivity (Table 3). According to sequence optimization, Romand et al. designed the human IGF-1 analog IGF-1Ea mut peptide, which reduced the degradation and the binding with the inhibitory IGF-binding protein, prevented the cutting of the Ea part, and improved its clinical efficacy (Romand et al. 2016).

**Table 3:** Vector optimization strategy of RTPs degradation in CHO cells

Cell type	Condition	Outcome	References
CHO-DUXB11	Sequence optimization, mutation, deletion	Eliminate amino acid that are prone to degradation	Dorai et al. 2011; Robert et al. 2009; Strohl 2018
CHO	Sequence optimization	Efficiently express IGF-1Ea and inhibit its degradation	Romand et al. 2016
CHO (Pro^−5^)	O/N-linked glycosylation	Reduced sensitivity to hydrolase	Salvi et al. 2022
CHO-K1SV GS-KO	Fc-fusion tag	Inhibit VEGFR1(D1-D3) degradation	Chakrabarti et al. 2016
CHO-S/CHO-DG44	HSA fusion	Improve r-protein stability	Ji et al. 2019; Ueda et al. 2020
CHO-K1SV GS-KO	Codon optimization	Reduced mRNA translation elongation	Knight et al. 2022; Xie et al. 2019

IGF-1, insulin-like growth factor 1; HSA, human serum albumin

Unlike mAbs, which show resistance to proteolytic activity, glycoproteins can be very sensitive to proteases and other degradation activities due to their relatively exposed three-dimensional structure. Therefore, recombinant sequences are designed to include one or more additional O- or N-linked glycosylation sites in the exposure domain of a particular glycoprotein to reduce sensitivity to hydrolases. The glycobase structure is used as a recognition sequence by various folding proteins and glycosidases, and these interactions used to induce the degradation. Taken the importance of glycosyl structure in ER for folding and degradation into account, the combination of manganese, uridine, and galactose can increase in galactosylation and sialylation in the early stages of the glycosylation pathway. While additional dexamethasone supplementation would significantly improve sialic acid terminal occupation. In addition, N-link glycosylation is essential for insulin-like growth factor 1 (IGF-1R) signal transduction in CHO cells, and loss of IGF-1R glycosylation through sequence optimization promotes receptor degradation (Salvi et al. 2022).

Fusion protein, produced by the fusion of genes encoding two or more proteins without affecting pharmacokinetics, is another strategy to extend the half-life of most protein drugs and delay the degradation, such as Fc fusion protein. VEGFR1(D1-D3)-Fc fusion protein was constructed in CHO-K1SV GS-KO cells, which contained extracellular domains 1, 2, and 3 of human VEGF receptor 1, and fused with the Fc region of IgG1. Protein degradation patterns are not the same in clones when cultured at low temperature (Chakrabarti et al. 2016). Over the past decade, a large number of genes have been fused into human serum albumin (HSA) in CHO cell (GIBCO, #A1155701), including interferon, erythropoietin, clotting factor IX and VIIa, hGH, TNF, and antibody fragments to improve the stability of the POIs (Ji et al. 2019; Ueda et al. 2020). High elongation induces folding errors and reduces fidelity, resulting in regions which are easily degraded/hydrolyzed (Xie et al. 2019). By means of codon optimization, different transcription sequences are designed for IgG Fc fusion protein expression, thereby changing the translation elongation rate of mRNA and effectively reducing protein fragmentation (Knight et al. 2022).

### Medium additive

Another way to control protease activity in cell culture is to add inhibitors or alternative substrates (Table 4), such as plant peptone or albumin to the medium (Li et al. 2021; Schauer et al. 2018; Zhang et al. 2023b). With developing quality by design (QbD) approach, the researchers revealed many physical and chemical factors that affect the mass of POIs, and screened for the unique substances at one or more stages of the RTP development process, to weaken degradation through combining with the properties of the target protein. In the process of large-scale antibody production, amino acid levels must be strictly controlled to achieve high productivity. Due to endoplasmic reticulum (ER) stress, the addition of cystine promotes ERAD and decreases cell viability and productivity. The simultaneous addition of tyrosine and cystine (3:3 ratio) decreased ERAD and increased mAb production by activating glutathione GSH metabolism, inhibiting ER stress and oxidative stress (Shibafuji et al. 2023).

**Table 4:** Medium additive strategy of RTPs degradation in CHO cells

Cell type	Condition	Outcome	References
CHO-DHFR	Iron citrate	Inhibit serine protease and metalloprotease	Clincke et al. 2011
CHO-DG44	Tyrosine and cystine	Improve GSH levels and decrease ERAD	Shibafuji et al. 2023
CHO-K1	Cyclodextrin	Protein stabilizer and inhibit degradation	Bognanni et al. 2021
CHO-K1	ABSEF, benzamide HCL, aprotinin, Batimastat	Inhibit host cell serine protease activity	Clarke et al. 2019; Clincke et al. 2011
CHO-K1SV GS-KO	Cocktail	Serine protease activity decreased	Chakrabarti et al. 2016
CHO-K1	Epoxomicin	Irreversible proteasome inhibitor	Knight et al. 2021
CHO-K1	MG-115	Proteasome inhibitor	Yamamoto et al. 2023
CHO-K1	MG-132	Inhibit proteasome	Knight et al. 2021
CHO	E-64, leupeptin	Inhibit lysosomes activity	Hishinuma et al. 2018
CHO-K1	S-sulfurine cysteine	Reduce antibody fragments	Seibel et al. 2017
CHO-K1SV GS-KO	PMSF and benzoylamine	Reduction fusion protein fragments	Knight et al. 2022
CHO-S	Pluronic VR F-68	Reduced filtration performance	Zacchi et al. 2020
CHO-K1/CHO-DUXB11	Sodium azide	Prevents riboflavin-induced photodegradation	Mantha et al. 2020
CHO-K1SV GS-KO	Cycloheximide	Slow down mRNA translation	Knight et al. 2022
CHO-S	Apilimod	Inhibit autophagy-induced degradation	Lu et al. 2023
CHO-K1	Leupeptin	Inhibited cathepsin B cleavage activity	Hu et al. 2022

ABSEF, 4-(2-aminoethyl) benzene sulfonyl fluoride hydrochloride; E64, cysteine proteases inhibitor E 64; PMSF, phenylmethanesulfonyl fluoride

Protein degradation is closely related to the degree of aggregation, and protein stabilizers, such as cyclodextrin added to the medium could reduce aggregation heterogeneity and thus inhibit degradation (Bognanni et al. 2021). In addition, the addition of ferric citrate to IFN-γ-producing CHO culture significantly inhibited proteolysis, indicating metalloproteinases presence in the culture. Serine proteinase aprotinin and 4-(2-aminoethyl) benzene sulfonyl fluoride hydrochloride (ABESF) have been shown to largely inhibit the proteolytic activity of gp120 protein production in CHO cells (Clarke et al. 2019; Clincke et al. 2011). The addition of protease inhibitor cocktail (serine and cysteine protease inhibitors) in culture increased cell viability with increasing culture days, compared to the control group without inhibitors (Chakrabarti et al. 2016). Trisulfides, a common modification in natural and recombinant mAbs of all IgG subtypes, are detected only in the inter-chain linkages, especially in light and heavy chains, and show vital effects on the aggregation and fragmentation of antibodies. Seibel et al. added S-sulfurine cysteine in the process of cell culture and helped to reduce the percentage of fragments produced by mAb (Seibel et al. 2017). During CHO-K1 cell culture, a decrease in the number of IgG1 HC Fc fusion protein fragments was observed when treated with 2 mM proteasome inhibitor phenylmethanesulfonyl fluoride (PMSF) and 4 mM benzoylamine (Knight et al. 2022).

Pluronic VR F68 (PF-68) is another additive used to protect cells from cleavage effects, and increasing concentration of PF-68 negatively affects filtration performance, prolongs the residence time of RTPs in bioreactors, improves protein degradation, and decreases product quality (Zacchi et al. 2020). Sodium azide is a well-known scavenger of reactive oxygen species and has been shown to resist the photooxidation pathways. The addition of 0.02% sodium azide or 1 mM glutathione to mAb samples significantly prevented riboflavin-induced photodegradation of mAb1. Sodium azide also protects mAb1 from ascorbate-mediated monomer loss (Mantha et al. 2020). S-Sulfocysteine is a cysteine derivative, which used as an antioxidant and added to cell medium to reduce the percentage of antibody fragments produced, reducing the trisulfide bonds between the medium and light chains, and product heterogeneity (Hecklau et al. 2016; Seibel et al. 2017).

Cycloheximide is a well-established chemical agent that inhibits eukaryotic translation elongation, slowing mRNA translation at concentrations of 0.025 to 0.05 μg/ml, resulting in reduced elongation rates of peptides and fragment levels of fusion proteins (Knight et al. 2022). Differential transcriptomics showed that meltrin β and furin expression were increased in the high Fc fusion proteolytic group. The proteases meltrin β and furin can specifically recognize arginine residues and lyse them. Adding furin inhibitor I (a family of proprotein convertases that specifically recognize proprotein convertases) could increase the integrity of IgG4 Fc fusion protein by 33–39%, compared to untreated samples (Clarke et al. 2019). Autophagy pathway has a wide range of physiological effects and is a highly conserved lysosomal-mediated degradation mechanism in eukaryotic cells. Our previous studies found that the small molecule compound apilimod-treated CHO cells significantly inhibited autophagy and increased the yield of RTPs (Lu et al. 2023).

Some proteins remain difficult-to-express (DTE) in the CHO cell expression system. DTE proteins could induce the unfolded protein reaction (UPR), ERAD, and proteasome degradation processes, when cells express the DTE proteins and exceed the cell folding and modification capacity. Especially, when DTE mAbs impede secretion, these molecules are degraded by proteasomes in order to maintaining protein quality and enabling amino acid cycling (Mathias et al. 2020). In the cell pool construction process, the irreversible inhibitor epoxomycin and another reversible inhibitor MG-132 were added to the cell culture medium to inhibit the proteasome activity and reduce protein degradation, then improving the cell production capacity (Knight et al. 2021). Hu et al. found that leupeptin (a serine, threonine, and cysteine proteases inhibitor) can effectively inhibited cathepsin B-mediated cleavage activity of the target bsAb fragmentation (Hu et al. 2022).

Although some small additives decrease degradation and improve the production efficiency of recombinant proteins, different small additives may have non-specific effects on CHO cell lines growth. Thus, the mechanism by which small additives affect degradation is complex and may remain to be further elucidated.

### Culture process optimization and other strategies

Product quality analysis of the cell line revealed that considerable protein degradation, reducing the proportion of full-length RTPs production, resulting in heterogeneity. Preliminary studies have shown that changing culture parameters, such as increasing cell culture pH or lowering temperature, could reduce product degradation (Table 5), while an increase in osmotic pressure was associated with an increase in product degradation (Clarke et al. 2019). Other methods of reduced degradation of RTPs include medium optimization, and early harvest of products to reduce the amounts of proteases released into the medium from non-viable cells (Dorai et al. 2011; Kaur et al. 2023). The effects of pH variation on RTPs are multifactorial, including changes in the titer and quality of mAbs, N-glycosylation, protein aggregation, and charge variation (Paul et al. 2018; Xie et al. 2016). Zheng et al. showed that both pH and buffer composition play a role in controlling the degradation behavior of mAbs; however, pH is more significant. Under low temperatures, pH4.5 leads to the most unstable structure of the antibody and a greater degree of fragmentation (Zheng et al. 2017).

**Table 5:** Culture process strategy of RTPs degradation in CHO cells

Cell type	Condition	Outcome	References
CHO-K1SV GS-KO	Osmotic pressure	High osmotic pressure promotes degradation	Clarke et al. 2019
CHO	pH4.5	Promote mAb fragment	Zheng et al. 2017
CHO-S	37 to 35–29 °C	Inhibit proteolytic enzyme binds to cleavage sites	Torres and Dickson 2022
CHO-K1SV GS-KO	Temperature from 37 to 35–29 °C; 32 °C; 30 °C	Reduced total protease expression and activity; improve protein folding	Bryan et al. 2021; Chakrabarti et al. 2016; Henry et al. 2018; Knight et al. 2022; Torres et al. 2021
CHO (TF 70R)	Low temperature (28–34 °C)	Inhibit degradation through affecting N-glycosylation	Vergara et al. 2018
CHO-K1	31 °C with valeric acid in perfusion	Inhibit VIII factor degradation	Coronel et al. 2020
CHO-K1	Perfusion culture	Changed the residence time inside the bioreactor	Bielser et al. 2019; Gomez et al. 2020
CHO-K1	Intensified perfusion	Decrease aggregation and fragmentation	Qin et al. 2022

MMP-9/12, matrix metalloproteinases-9/12

Culture temperature has been extensively studied and applied to CHO cell-based RTPs production (Zhu et al. 2023). Subphysiological temperature slows down translation by affecting a number of cellular responses, including phosphorylation of translation extension factor eEF2, leading to a slowing of peptide elongation. The inhibited degradation is closely related to the decreased protein synthesis rate and the improvement of folding caused by low temperature. The reduction from the standard 37 to 35–29 °C improved the cell-specific productivity (Torres et al. 2018), and the mechanisms are that low temperature increased mRNA stability, and reduced proteolytic enzymes recognize cleavage sites, resulting in a large amount of recombinant mRNA and contributes to production (Torres and Dickson 2022). Chakrabarti et al. showed that simply lowering the culture temperature from 37 to 30 °C was the best way to reduce protein degradation (Chakrabarti et al. 2016).

In industrial production, lower culture temperature significantly affected cell growth and cell productivity of CHO cells (Nguyen et al. 2020; Yang et al. 2022). For antibody expression, the decrease in temperature significantly affected N-glycosylation, showing less processed glycosylation structures in the stable region of mAbs and affecting degradation (Vergara et al. 2018). In the process of CHO cell fed-batch culture expressing human erythropoietin (hEPO), low temperature culture changed the expression patterns of the UPR/ERAD-specific genes, such as E3 ubiquitin-protein ligase synoviolin 1 and ER degradation enhancing alpha-mannosidase like protein 3. These proteins are involved in the folding and processing of proteins in the ER and the degradation of RTPs to enhance the production of chimeric fusion proteins in CHO cells (Torres et al. 2021).

Traditional fed-batch cell culture has become a common technique for RTP production and the dominant method for mAs and other RTPs production (MacDonald et al. 2022a; Schulze et al. 2022; Xu et al. 2023). The feed is a highly concentrated nutrient solution that causes an increase in osmotic pressure within the culture. Romanova et al. performed proteomics research and found that most of the genes involved in proteolysis include C1, E3 ubiquitin protein ligase, ubiquilin 2, and cytotoxic T lymphocyte-associated protein 2 α/β, were downregulated from the 6th to 8th day of batch culture with feeding, confirmed that excessive fed supplementation of CHO cells (ATCC: CRL-12445), enhanced protein synthesis, and inhibited protein degradation (Romanova et al. 2022).

Perfusion (PER) culture is a continuous culture mode, in which fresh medium is continuous to be added into the bioreactor and the product is continuously harvested. Therefore, PER culture is a relatively new technique to maintain cell viability and harvest high-quality proteins (Liang et al. 2023; Schwarz et al. 2023). PER culture also provides a narrower residence time distribution for products, especially unstable RTPs like FVIII, limiting exposure to extracellular enzymes (e.g., proteases, glycosidases, sialidases) as well as the extracellular environment (pH, temperature) (Coronel et al. 2020). Increasing the control of cell environment, cell metabolism, and product environment could reduce the heterogeneity of protein aggregation and fragmentation (Karst et al. 2018). For example, Bielser et al. recently reported that the cleavage of a fusion protein was as high as 9% during the fed-batch process, while the cleavage was reduced to 1.5% during the PER process (Bielser et al. 2019). Compared with mAbs, bispecific antibodies (bsAbs) are DTE and more prone to physical and chemical degradation and fragmentation, resulting in higher levels of protein-modified residues in cell culture. Intensified PER cell culture is a better option than traditional fed-batch culture, especially for complex molecules like bsAbs. Consistent with PER culture, the quality of the bispecial products was continuously improved and the level of shear fragmentation was reduced (by 75%) (Gomez et al. 2020). Compared with the traditional fed-batch model, Qin et al. cultured CHO cells with intensive PER to express bsAbs, and the 23-day intensive perfusion process significantly reduced aggregation and fragmentation (Qin et al. 2022).

Studies have confirmed that valeric acid (VA) affects glycosylation and is conducive to terminal galactosylation of mAbs (Park et al. 2016). In addition, Wolf et al. lowered the culture temperature and added 1 mM VA to the medium to form a chemical growth inhibition (CGI) to detect the effect on the quality of antibody expression in mammalian cell PER culture. The results showed that CGI reduced antibody aggregation (2.5–3.8%) and fragmentation (1.5–2.2%), significantly lower than the control group (4.1%) (Wolf et al. 2019).

### Purification process

Inhibition of protease is important for the hydrolysis of RTPs during cell culture. Optimization strategies for protein purification processes (Table 6) are also indispensable for eliminating protease activity and inhibiting degradation (Kim et al. 2023). Cui et al. used caprylate wash buffer to remove residual cathepsin D and the associated fragmentation in mAb (Cui et al. 2019). Hu et al. optimized protein A chromatography by adding sodium caprylate to the wash buffer, and effectively removed HCPs including endogenous protease(s) that are responsible for target antibody fragmentation (Hu et al. 2021). Consistently, Yang et al. added wash buffer with tris and arginine at pH 8.5 to enhance the residual host cell protease(s) removal capability in the purification process and consequently eliminate fragment of mAb (Yang et al. 2019). Purification strategies could be also implemented to remove lipases, such as using lectins to remove high mannose HCP glycoforms (Hecht et al. 2022). Cathepsin L exhibits proteolytic activity over a mildly acidic pH range of 3.5–6.0, making it a concern for purification of RTPs. Luo et al. combined anion exchange with hydrophobic interactions chromatography, named Capto™ adhere, and reduced cathepsin L to nondetectable levels (Luo et al. 2019). Moreover, Zhang et al. performed activity-based protein profiling to separate and identify liver carboxylesterase B-1 like and carboxylesterase 1 like and inhibit the rapid polysorbate80 degradation in the purified mAb (Zhang et al. 2020).

**Table 6:** Purification strategy of RTPs degradation in CHO cells

Condition	Outcome	References
Caprylate wash buffer	Remove residual cathepsin D	Cui et al. 2019
Add sodium caprylate to wash buffer	Removed endogenous protease(s)	Hu et al. 2021
Add tris and arginine to wash buffer	Enhance the residual host cell protease(s) removal capability	Yang et al. 2019
Using lectins	remove lipases	Hecht et al. 2022
Combined anion exchange with hydrophobic interactions chromatography	Reduced cathepsin L to nondetectable levels	Luo et al. 2019
Performed activity-based protein profiling	Separate and identify liver carboxylesterase	Zhang et al. 2020
Surface-imprinted polymer particles	Removal of the target protease	Pluhar et al. 2015
Thermolysin-imprinted beads	Removal of MMP-9 and MMP-12	Schauer et al. 2018
Novel resins	Improved capacity to bind to LPl, cathepsin B, MMP-9, MMP-19, and sialidase-1	Lavoie et al. 2019; Lavoie et al. 2020

MMP-9/12/19, matrix metalloproteinases-9/12/19

Based on chromatography and the use of affinity matrices, such as benzoylamine agarose, the degradation of products by proteases can be removed during CHO cell production. Pluhar et al. prepared protease-imprinted polymers by micro-emulsion polymerization and selectively removed target proteases from supernatant (Pluhar et al. 2015). Moreover, Schauer et al. further optimized the method by adopting thermolysin imprinted beads and binding matrix MMP-9 and MMP-12 in the supernatant of CHO cell culture to reduce the degradation of RTPs (Schauer et al. 2018). Lavoie RA et al. found that novel resins showed improved capacity at high salt concentration to bind to HCPs, including LPl, cathepsin B, MMP-9, MMP-19, and sialidase-1, which degrade mAbs or affect mAb stability (Lavoie et al. 2019, 2020).

## Summary and prospects

**Fig. 1:**
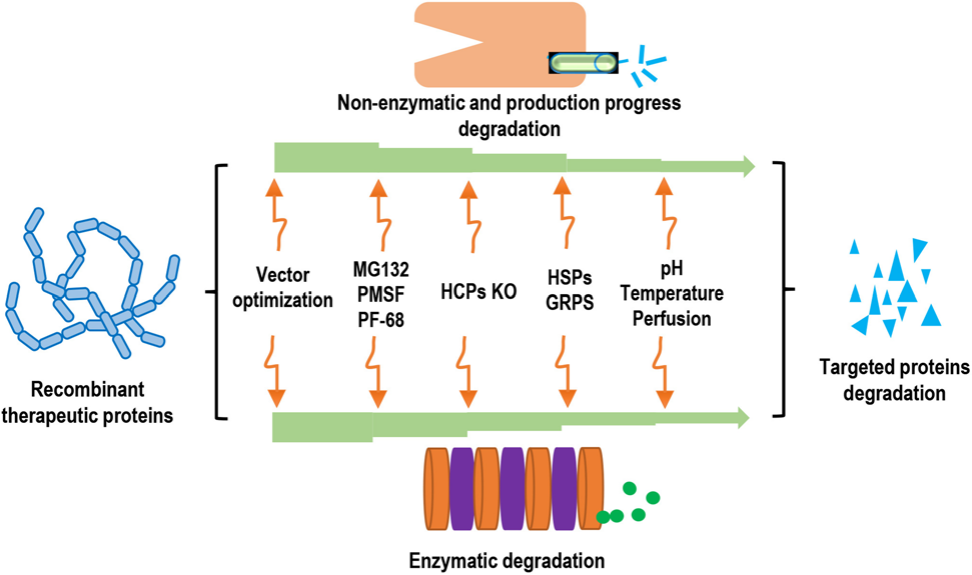
The influencing factors of recombinant therapeutic proteins degradation in CHO cells and the main overcome strategies are briefly described. Overcome strategies are in the middle of the orange line with arrows

Degradation could inhibit the functions of RTPs and reduce quality, resulting in lack of immune function. At present, extensive progresses have been made in improving and maintaining the quality of CHO cell-expressed proteins by inhibiting degradation. There are several strategies to decrease degradation, including vector optimization, medium additive, gene knockout, and culture parameter adjustment (Fig. 1). However, mechanisms underlying regulation of degradation are complex, and particularly, there are many kinds of endogenous proteases and their regulatory mechanisms are more complex and play a crucial role in the process of RTPs degradation.

In addition to the host difficult-to-remove hydrolase, increasing RTP expression levels will trigger the stress response of host cells, and the cell folding and modification capacity are exceeded, which would induce the UPR, ERAD, and proteasome degradation processes. Future research is still needed to screen key genes that enhance CHO cell folding and secretory ability. The emergence of CRISPR/Cas9 technology opens up new ways to engineer CHO cell lines and improve protein production and quality. Combined gene editing methods with the wide application of multi-omics analysis (Lee et al. 2021), artificial intelligence (Zhang et al. 2023a), and other methods to screen key factors and design expression sequences that resist degradation, the mechanisms of cell degradation will be further clarified, which may guide the optimization of cell engineering strategies.
